# Supplementary material for: Dynamic chromatin regulatory landscape of human CAR T cell exhaustion
Source: Proc Natl Acad Sci U S A. 2021 Jul 20;118(30):e2104758118. doi: 10.1073/pnas.2104758118 (PMC8325267; doi:10.1073/pnas.2104758118)
Supplement: Supplementary File [file pnas.2104758118.sapp.pdf]

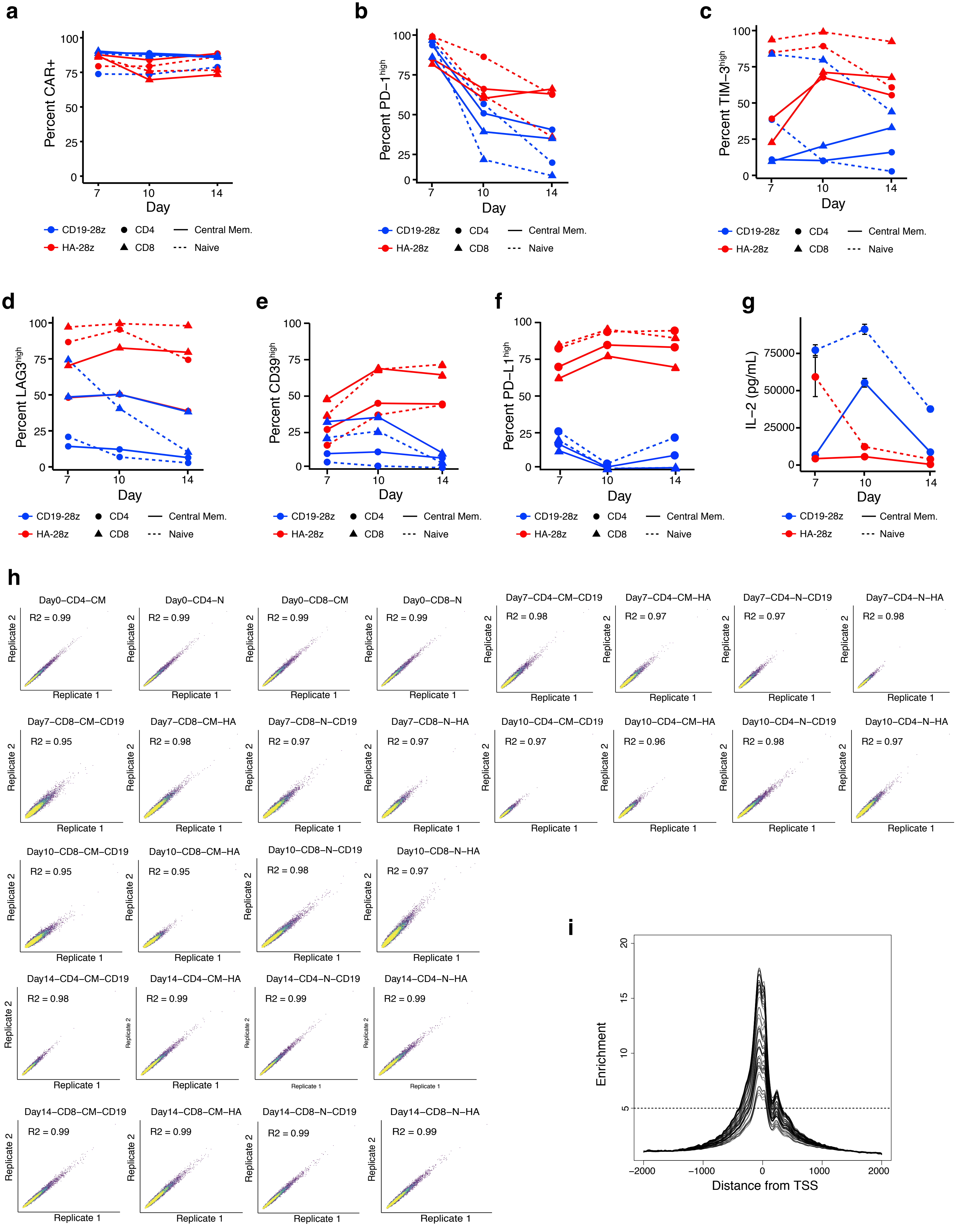
a
Percent CAR+
Day
b
Percent PD−1high
c
Percent TIM−3high
d
Percent LAG3high
e
Percent CD39high
f
Percent PD−L1high
g
IL−2 (pg/mL)
CD19-28z
HA-28z
CD4
CD8
Central Mem.
Naive
h
Day0−CD4−CM
R2 = 0.99
Day0−CD4−N
R2 = 0.99
Day0−CD8−CM
R2 = 0.99
Day0−CD8−N
R2 = 0.99
Day7−CD4−CM−CD19
R2 = 0.98
Day7−CD4−CM−HA
R2 = 0.97
Day7−CD4−N−CD19
R2 = 0.97
Day7−CD4−N−HA
R2 = 0.98
Day7−CD8−CM−CD19
R2 = 0.95
Day7−CD8−CM−HA
R2 = 0.98
Day7−CD8−N−CD19
R2 = 0.97
Day7−CD8−N−HA
R2 = 0.97
Day10−CD4−CM−CD19
R2 = 0.97
Day10−CD4−CM−HA
R2 = 0.96
Day10−CD4−N−CD19
R2 = 0.98
Day10−CD4−N−HA
R2 = 0.97
Day10−CD8−CM−CD19
R2 = 0.95
Day10−CD8−CM−HA
R2 = 0.95
Day10−CD8−N−CD19
R2 = 0.98
Day10−CD8−N−HA
R2 = 0.97
Day14−CD4−CM−CD19
R2 = 0.98
Day14−CD4−CM−HA
R2 = 0.99
Day14−CD4−N−CD19
R2 = 0.99
Day14−CD4−N−HA
R2 = 0.99
Day14−CD8−CM−CD19
R2 = 0.99
Day14−CD8−CM−HA
R2 = 0.99
Day14−CD8−N−CD19
R2 = 0.99
Day14−CD8−N−HA
R2 = 0.99
Replicate 1
Replicate 2
i
Enrichment
Distance from TSS

Fig. S1. Donor-derived CAR T cell phenotyping and Omni-ATAC-seq quality control metrics. (A) Surface expression of CAR on transfected T cell populations. (B-G) Surface expression and cytokine secretion of markers of T cell exhaustion throughout the CAR T cell maturation time course. (H) All samples demonstrate high replicate concordance. (I) All samples demonstrate high enrichment of genome alignment to transcription start sites.

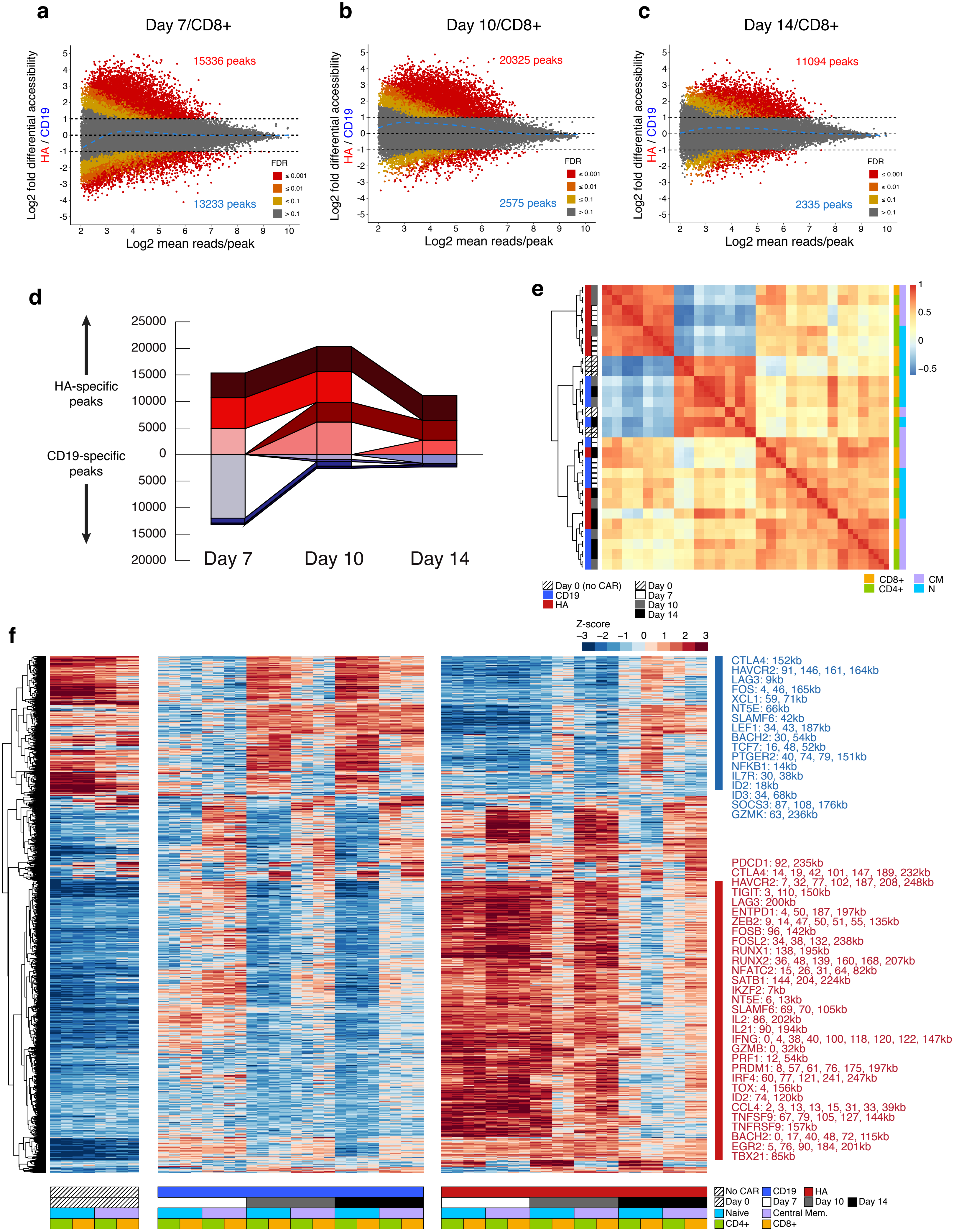
a
Day 7/CD8+
15336 peaks
13233 peaks
b
Day 10/CD8+
20325 peaks
2575 peaks
c
Day 14/CD8+
11094 peaks
2335 peaks
Log2 fold differential accessibility
HA / CD19
Log2 mean reads/peak
FDR
≤ 0.001
≤ 0.01
≤ 0.1
> 0.1
d
HA-specific peaks
CD19-specific peaks
25000
20000
15000
10000
5000
0
5000
10000
15000
20000
Day 7
Day 10
Day 14
e
1
0.5
0
−0.5
Day 0 (no CAR)
CD19
HA
Day 0
Day 7
Day 10
Day 14
CD8+
CD4+
CM
N
f
Z-score
−3 −2 −1 0 1 2 3
CTLA4: 152kb
HAVCR2: 91, 146, 161, 164kb
LAG3: 9kb
FOS: 4, 46, 165kb
XCL1: 59, 71kb
NT5E: 66kb
SLAMF6: 42kb
LEF1: 34, 43, 187kb
BACH2: 30, 54kb
TCF7: 16, 48, 52kb
PTGER2: 40, 74, 79, 151kb
NFKB1: 14kb
IL7R: 30, 38kb
ID2: 18kb
ID3: 34, 68kb
SOCS3: 87, 108, 176kb
GZMK: 63, 236kb
PDCD1: 92, 235kb
CTLA4: 14, 19, 42, 101, 147, 189, 232kb
HAVCR2: 7, 32, 77, 102, 187, 208, 248kb
TIGIT: 3, 110, 150kb
LAG3: 200kb
ENTPD1: 4, 50, 187, 197kb
ZEB2: 9, 14, 47, 50, 51, 55, 135kb
FOSB: 96, 142kb
FOSL2: 34, 38, 132, 238kb
RUNX1: 138, 195kb
RUNX2: 36, 48, 139, 160, 168, 207kb
NFATC2: 15, 26, 31, 64, 82kb
SATB1: 144, 204, 224kb
IKZF2: 7kb
NT5E: 6, 13kb
SLAMF6: 69, 70, 105kb
IL2: 86, 202kb
IL21: 90, 194kb
IFNG: 0, 4, 38, 40, 100, 118, 120, 122, 147kb
GZMB: 0, 32kb
PRF1: 12, 54kb
PRDM1: 8, 57, 61, 76, 175, 197kb
IRF4: 60, 77, 121, 241, 247kb
TOX: 4, 156kb
ID2: 74, 120kb
CCL4: 2, 3, 13, 13, 15, 31, 33, 39kb
TNFSF9: 67, 79, 105, 127, 144kb
TNFRSF9: 157kb
BACH2: 0, 17, 40, 48, 72, 115kb
EGR2: 5, 76, 90, 184, 201kb
TBX21: 85kb
No CAR
CD19
HA
Day 0
Day 7
Day 10
Day 14
Naive
Central Mem.
CD4+
CD8+

Fig. S2. The dynamics of accessible chromatin loci over time identify loci with varying temporal control. (A-C) Differentially accessible chromatin loci (peaks) in CD8+ CAR T cells at 7, 10, and 14 days following CAR transfection. (D) Differentially accessible loci across time points, colored by which timepoints and the number of timepoints in which they are differentially accessible. Loci differentially accessible in HA-28z CAR T cells (red) are more numerous persist through multiple time points to a greater degree and are more numerous than the loci differentially accessible in CD19-28z CAR T cells (blue). (E) Sample clustering by Pearson correlation of global chromatin accessibility profiles. (F) Top 5000 most variable chromatin accessibility peaks across all samples colored by z-score of peak accessibility in each sample. Many of the peaks differentially accessible in either the CD19-28z or HA-28z CAR T cells appear proximal to known exhaustion-associated and T cell effector genes. Distances between peaks and select gene promoters are listed.

a

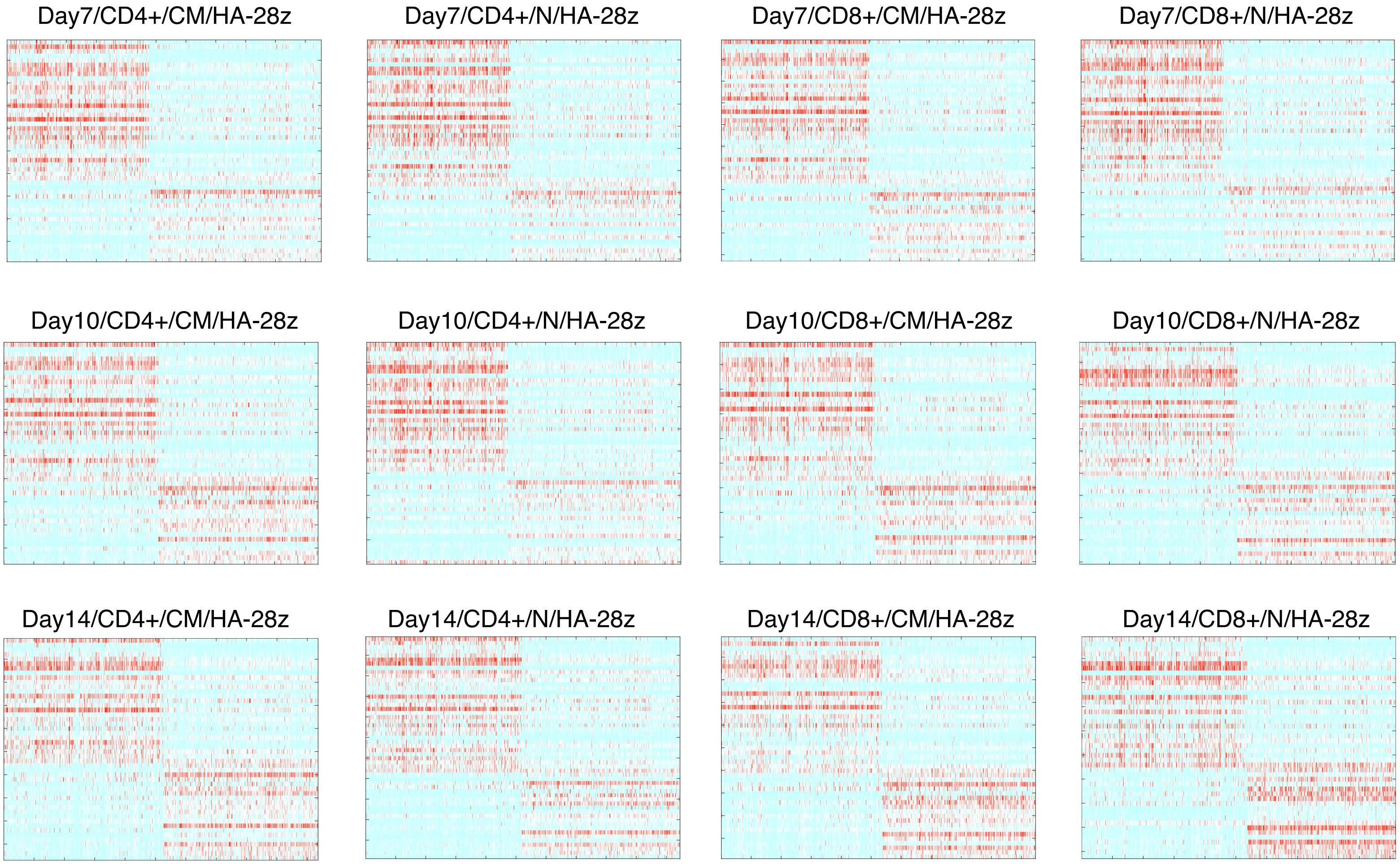
Day7/CD4+/CM/HA-28z
Day7/CD4+/N/HA-28z
Day7/CD8+/CM/HA-28z
Day7/CD8+/N/HA-28z
Day10/CD4+/CM/HA-28z
Day10/CD4+/N/HA-28z
Day10/CD8+/CM/HA-28z
Day10/CD8+/N/HA-28z
Day14/CD4+/CM/HA-28z
Day14/CD4+/N/HA-28z
Day14/CD8+/CM/HA-28z
Day14/CD8+/N/HA-28z

b

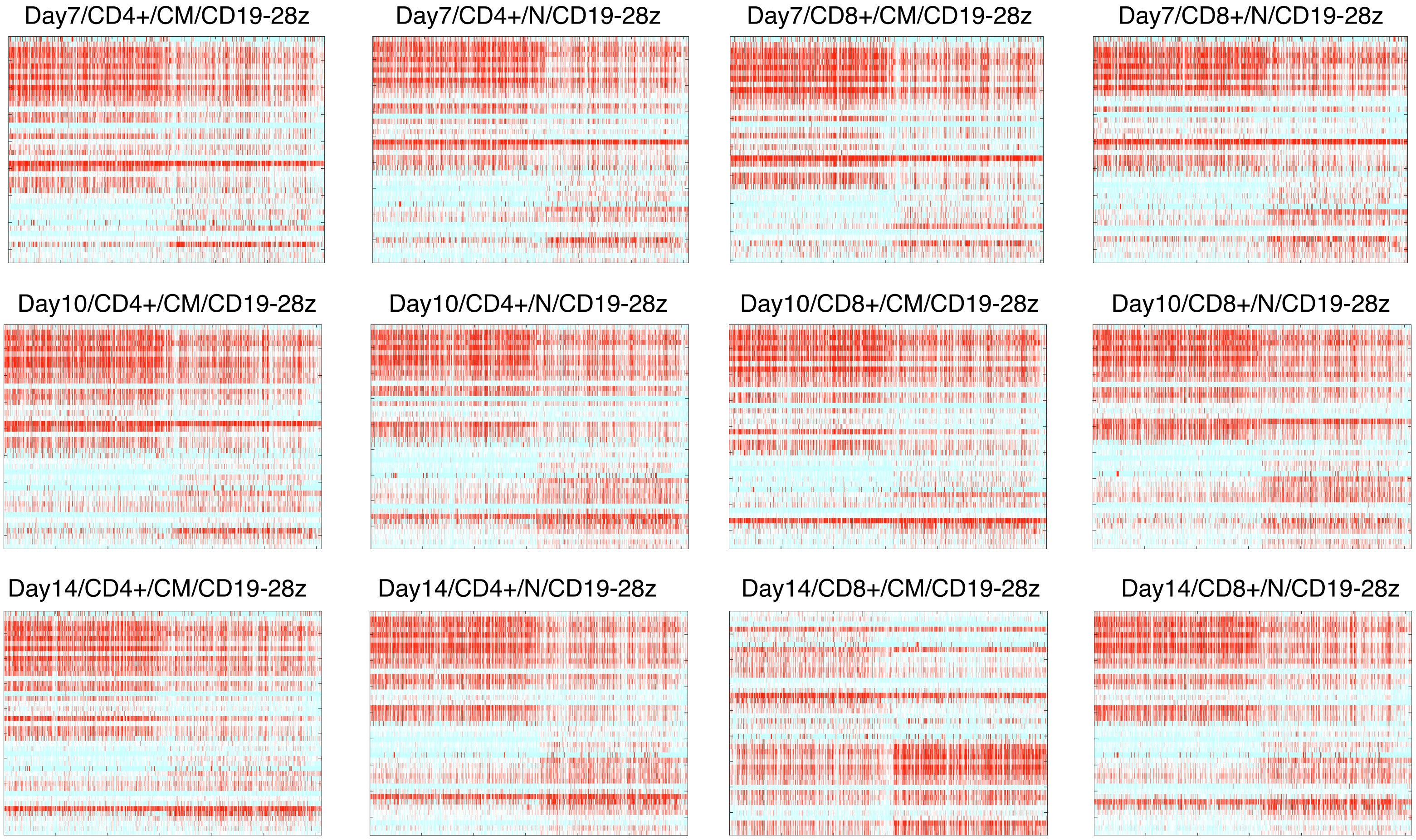
Day7/CD4+/CM/CD19-28z
Day7/CD4+/N/CD19-28z
Day7/CD8+/CM/CD19-28z
Day7/CD8+/N/CD19-28z
Day10/CD4+/CM/CD19-28z
Day10/CD4+/N/CD19-28z
Day10/CD8+/CM/CD19-28z
Day10/CD8+/N/CD19-28z
Day14/CD4+/CM/CD19-28z
Day14/CD4+/N/CD19-28z
Day14/CD8+/CM/CD19-28z
Day14/CD8+/N/CD19-28z

Fig. S3. Transcription factor-gene dynamics demonstrate coregulated modules in exhausted CAR T cells. PECA2 coregulation score heatmaps for all (A) HA-28z and (B) CD19-28z CAR T cell samples, merging replicates, indicating the strength of correlation between transcription and accessibility dynamics across samples.

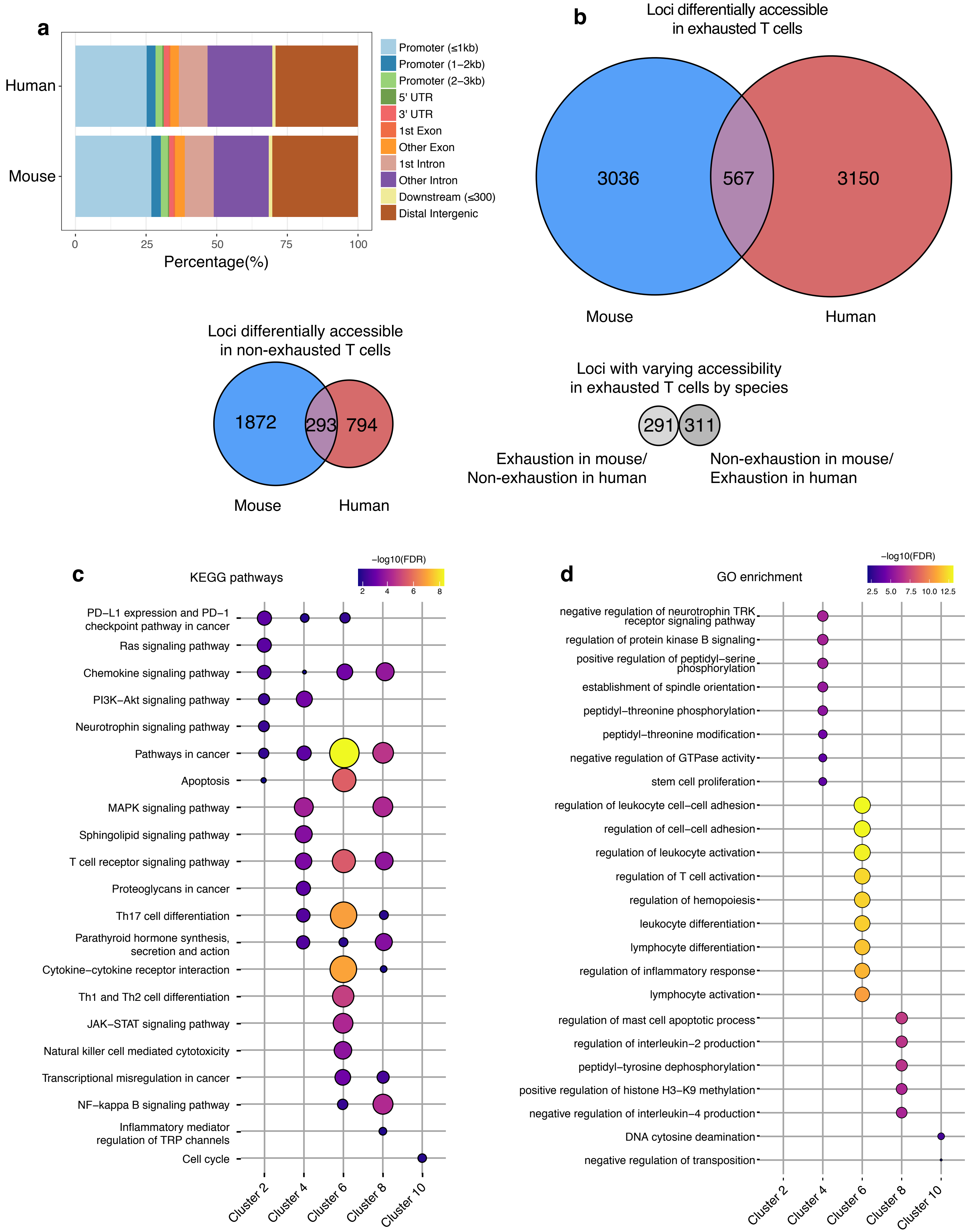
a
Human
Mouse
Promoter (≤1kb)
Promoter (1–2kb)
Promoter (2–3kb)
5' UTR
3' UTR
1st Exon
Other Exon
1st Intron
Other Intron
Downstream (≤300)
Distal Intergenic
0 25 50 75 100
Percentage(%)
b
Loci differentially accessible in exhausted T cells
3036
567
3150
Mouse
Human
Loci differentially accessible in non-exhausted T cells
1872
293
794
Mouse
Human
Loci with varying accessibility in exhausted T cells by species
291
311
Exhaustion in mouse/ Non-exhaustion in human
Non-exhaustion in mouse/ Exhaustion in human
c
KEGG pathways
−log10(FDR)
2 4 6 8
PD−L1 expression and PD−1 checkpoint pathway in cancer
Ras signaling pathway
Chemokine signaling pathway
PI3K−Akt signaling pathway
Neurotrophin signaling pathway
Pathways in cancer
Apoptosis
MAPK signaling pathway
Sphingolipid signaling pathway
T cell receptor signaling pathway
Proteoglycans in cancer
Th17 cell differentiation
Parathyroid hormone synthesis, secretion and action
Cytokine−cytokine receptor interaction
Th1 and Th2 cell differentiation
JAK−STAT signaling pathway
Natural killer cell mediated cytotoxicity
Transcriptional misregulation in cancer
NF−kappa B signaling pathway
Inflammatory mediator regulation of TRP channels
Cell cycle
Cluster 2
Cluster 4
Cluster 6
Cluster 8
Cluster 10
d
GO enrichment
−log10(FDR)
2.5 5.0 7.5 10.0 12.5
negative regulation of neurotrophin TRK receptor signaling pathway
regulation of protein kinase B signaling
positive regulation of peptidyl−serine phosphorylation
establishment of spindle orientation
peptidyl−threonine phosphorylation
peptidyl−threonine modification
negative regulation of GTPase activity
stem cell proliferation
regulation of leukocyte cell−cell adhesion
regulation of cell−cell adhesion
regulation of leukocyte activation
regulation of T cell activation
regulation of hemopoiesis
leukocyte differentiation
lymphocyte differentiation
regulation of inflammatory response
lymphocyte activation
regulation of mast cell apoptotic process
regulation of interleukin−2 production
peptidyl−tyrosine dephosphorylation
positive regulation of histone H3−K9 methylation
negative regulation of interleukin−4 production
DNA cytosine deamination
negative regulation of transposition
Cluster 2
Cluster 4
Cluster 6
Cluster 8
Cluster 10

Fig. S4. Features of human and mouse dysfunctional T cell accessible chromatin. (A) Genomic features of the accessible chromatin loci in exhausted human HA-28z CAR T cells and chronically stimulated mouse T cells (from Sen et al., 2016). (B) Quantification of the ATAC-seq peaks in the T cells of each species associated with (1) exhausted T cells, (2) non-exhausted T cells, or (3) both exhausted and non-exhausted T cells with varying accessibility by species. (C) KEGG pathway enrichment and (D) Gene Ontology (GO) term enrichment for genes associated with the ATAC-seq peaks within clusters of exhaustion-accessible ATAC-seq peaks in mouse and/or human T cells.

Promoters that overlap ATAC-seq peaks (13,788)
HA-28z vs CD19-28z

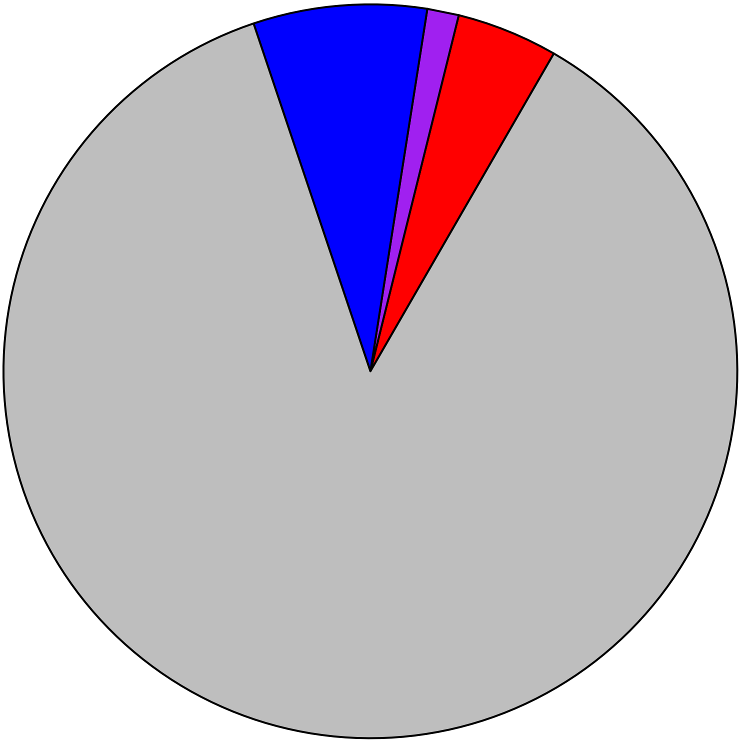

- Differential promoters, ATAC-only (615: 4.5%)
- Differential promoters, HiChIP-only (1053: 7.6%)
- Differential promoters, both ATAC + HiChIP (192: 1.4%)
- Non-differential promoters (11,928: 86.5%)

Fig. S5. Promoter regulation by chromatin accessibility and 3D chromosome looping. Of all gene promoters that exhibit chromatin accessibility by ATAC-seq in any CD19-28z or HA-28z CAR T cell sample, the proportions shown indicate promoters with differential ATAC-seq, HiChIP, both, or no differential signal between matched CD19-28z and HA-28z CAR T cells.

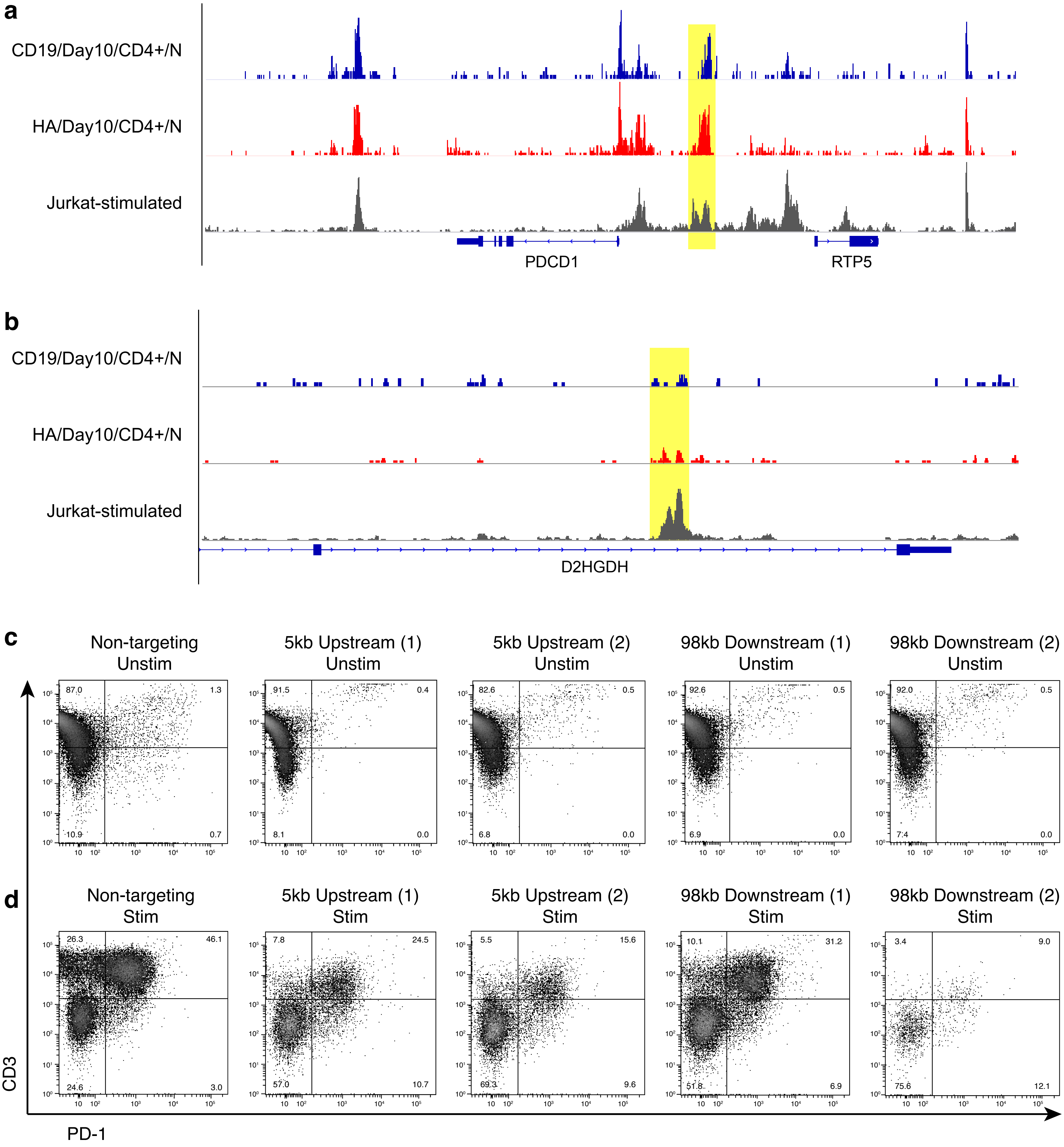

a
CD19/Day10/CD4+/N
HA/Day10/CD4+/N
Jurkat-stimulated
PDCD1
RTP5
b
CD19/Day10/CD4+/N
HA/Day10/CD4+/N
Jurkat-stimulated
D2HGDH
c
Non-targeting Unstim
5kb Upstream (1) Unstim
5kb Upstream (2) Unstim
98kb Downstream (1) Unstim
98kb Downstream (2) Unstim
87.0
1.3
10.9
0.7
91.5
0.4
8.1
0.0
82.6
0.5
6.8
0.0
92.6
0.5
6.9
0.0
92.0
0.5
7.4
0.0
d
Non-targeting Stim
5kb Upstream (1) Stim
5kb Upstream (2) Stim
98kb Downstream (1) Stim
98kb Downstream (2) Stim
26.3
46.1
24.6
3.0
7.8
24.5
57.0
10.7
5.5
15.6
69.3
9.6
10.1
31.2
51.8
6.9
3.4
9.0
75.6
12.1
CD3
PD-1

Fig. S6. The Jurkat T cell line shares regulatory loci with stimulated and exhausted CAR T cells. (A) Accessible chromatin sequencing alignment tracks of Day 10 CD19-28z, Day 10 HA-28z CAR T cells, and stimulated Jurkat cells (from Brignall et al., 2017) at the PDCD1 promoter locus and (B) 98 kb downstream from the PDCD1 transcription start site. (C) Cell surface expression of CD3 and PD-1 in unstimulated and (D) IL-2/CD3/CD28-stimulated Jurkat cells treated with non-targeting CRISPR/Cas9 RNPs, RNP pairs targeting the exhaustion-associated accessible chromatin locus 5 kb upstream of the PDCD1 promoter, or RNP pairs targeting 98 kb downstream of PDCD1 following 14 days of *in vitro* culture.

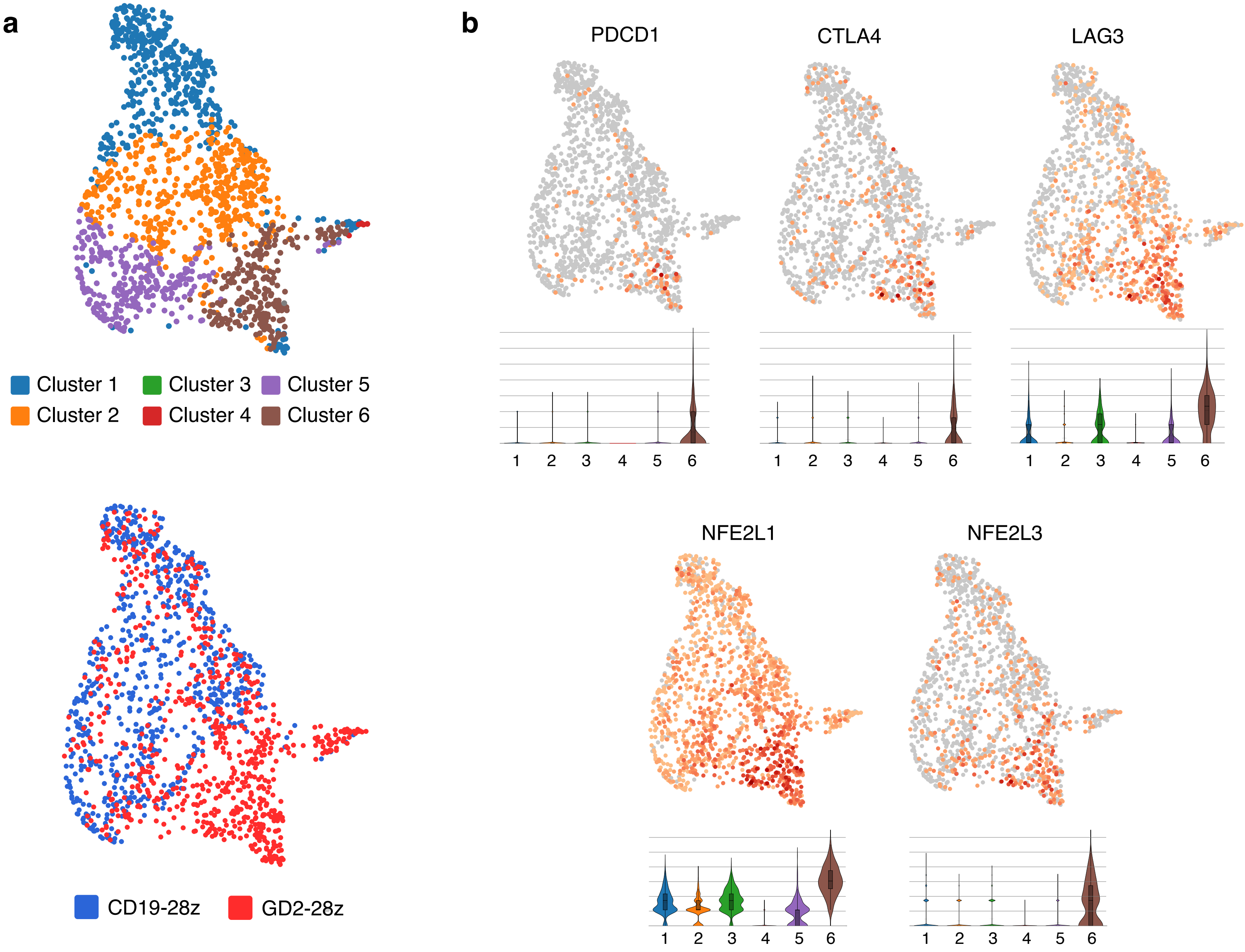
a
b
Cluster 1
Cluster 2
Cluster 3
Cluster 4
Cluster 5
Cluster 6
CD19-28z
GD2-28z
PDCD1
CTLA4
LAG3
NFE2L1
NFE2L3
1 2 3 4 5 6

Fig. S7. NFE2L1/NFE2L3 are co-expressed with markers of exhaustion. (A) UMAP dimensionality reduction of single-cell RNA-seq on non-exhausted CD19-28z and exhausted HA-28z CAR T cells (from Lynn et al., 2019) identifies a cluster of cells unique to the exhausted CAR T cell population. (B) Inhibitory receptor markers of exhaustion (PD-1, CTLA4, LAG3) and NFE2L1/3 are expressed in the exhaustion-specific cell cluster.

Table S1

TF	P-value	FDR	Fold	Score
NFE2L1	1.00E-321	7.15E-318	4.07071	35.9306
NFE2L3	1.81E-299	6.48E-297	3.65043	32.8818
POU2F2	2.16E-163	1.71E-161	4.1104	25.7063
TGIF1	1.59E-149	1.03E-147	3.67828	23.252
MAFF	1.11E-176	1.59E-174	2.65659	21.4874
CEBPB	8.27E-125	4.22E-123	3.30071	20.0978
JUND	2.19E-87	6.25E-86	3.98377	18.4237
ETS2	5.76E-89	1.79E-87	3.67791	17.8619
EGR3	4.44E-85	1.17E-83	3.84408	17.8547
TEAD4	4.11E-80	9.79E-79	3.82674	17.2777
RXRA	2.62E-74	5.21E-73	3.7404	16.4429
ETV4	1.85E-78	4.13E-77	3.52092	16.3995
FOXP4	1.46E-67	2.49E-66	3.98709	16.173
TGIF2	1.48E-102	5.58E-101	2.60241	16.1524
ARID3B	3.84E-89	1.25E-87	2.80478	15.6124
NFIC	7.47E-119	3.34E-117	1.94064	15.0346
NFAT5	4.39E-64	6.27E-63	3.5126	14.7815
FOSL1	5.25E-64	7.36E-63	3.49488	14.7359
ATF3	4.23E-65	6.30E-64	3.3094	14.4622
FOSL2	1.23E-60	1.49E-59	3.43656	14.2184

Table S1. Top 20 transcription factors identified by EnrichTF for regulatory function in Day 7 HA-28z CAR T cells differentially expressed in HA-28z CAR T cells. Table entries are listed by TF score, with each TF's enrichment one-sample t-test p-value, false- discovery rate (FDR), and fold-change compared to EnrichTF-identified enrichment in the matched Day 7 CD19-28z CAR T cell populations.

Table S2

TF	P-value	FDR	Fold	Score
NFE2L3	1.46E-159	1.05E-156	5.79589	30.0674
NFE2L1	1.35E-121	3.23E-119	5.58768	25.7311
ARID3B	2.13E-80	1.69E-78	5.36535	20.4272
TEAD4	5.82E-69	2.60E-67	5.24	18.679
MAFF	5.30E-88	4.74E-86	3.46677	17.1988
TGIF2	7.32E-69	3.08E-67	4.3253	16.9612
FOSL1	1.34E-47	2.39E-46	6.1492	16.7493
ETV4	3.36E-45	5.33E-44	6.33662	16.5591
BATF3	3.04E-44	4.63E-43	6.3196	16.3565
RXRA	1.50E-43	2.19E-42	6.22599	16.1052
NFIL3	4.22E-50	8.39E-49	5.38664	16.0925
THAP1	1.18E-56	2.82E-55	4.3853	15.4667
FOXB1	2.64E-68	9.43E-67	3.24124	14.6289
JUNB	4.40E-38	4.84E-37	5.66119	14.3383
ZEB1	8.57E-39	1.00E-37	5.38893	14.1202
TGIF1	1.55E-54	3.57E-53	3.32812	13.2117
SOX4	7.80E-31	6.64E-30	5.56854	12.7467
FOXM1	2.50E-59	6.61E-58	2.84122	12.746
FOXP4	2.32E-28	1.71E-27	5.32742	11.9414
CEBPB	1.20E-46	1.99E-45	3.02766	11.6335

Table S2. Top 20 transcription factors identified by EnrichTF for regulatory function in Day 10 HA-28z CAR T cells differentially expressed in HA-28z CAR T cells. Table entries are listed by TF score, with each TF's enrichment one-sample t-test p-value, false- discovery rate (FDR), and fold-change compared to EnrichTF-identified enrichment in the matched Day 10 CD19-28z CAR T cell populations.

Table S3

TF	D7-Fold	D10-Fold	D7-rank	D10-rank	Rank diff
NFE2L1	4.07070554	4.4578926	1	28	-27
FOXP4	3.98709444	5.04767828	2	22	-20
JUND	3.98376961	4.37279681	3	31	-28
EGR3	3.84408123	8.93906021	4	1	3
TEAD4	3.82674395	7.11165196	5	10	-5
RXRA	3.74039597	8.44084401	6	5	1
ARID5A	3.71299883	3.80964798	7	36	-29
TGIF1	3.67828431	3.68650483	8	38	-30
ETS2	3.67790674	3.51675332	9	41	-32
NFE2L3	3.65043038	8.41625773	10	6	4
EGR2	3.57333936	6.05721393	11	13	-2
CREM	3.56820818	5.46508728	12	19	-7
HIF1A	3.53286047	4.38379705	13	30	-17
ETV4	3.52092291	8.1082206	14	7	7
NFAT5	3.51260111	5.57440167	15	18	-3
FOSL1	3.4948784	8.67430025	16	3	13
BATF3	3.43956699	8.66950464	17	4	13
FOSL2	3.43656302	4.87522345	18	25	-7
ATF3	3.30939907	6.43612335	19	12	7
CEBPB	3.30070671	2.52404261	20	49	-29
JUNB	3.2060993	7.576229	22	9	13
ARID3B	2.80478197	8.86836629	32	2	30
SOX4	2.70233744	7.609375	35	8	27

Table S3. Top EnrichTF differentially active transcription factors at Day 7 between HA-28z CAR T cells and CD19-28z CAR T cells, compared to Day 10 CAR T cells. For each TF, the fold-change in EnrichTF enrichment is listed for Day 7 and Day 10 samples, comparing HA-28z and CD19-28z CAR T cells, along with the respective rank for the fold- change quantity for each. Red rows indicate TFs with a Day 10-enrichment rank higher than the Day 7 rank.

Dataset S1. (separate file)

The 5000 most differential ATAC-seq peaks across all T cell populations. For each peak (row), a unique name, hg19 genome coordinate, and regularized log-transformed accessibility score averaging replicates are provided.

Dataset S2. (separate file)

Target gene (TG) module assignment for clustered TF/target gene interactions called by EnrichTF/PECA2. Each target gene identified in the interaction matrix fell into one of two TF/TG modules by hierarchical clustering. The vast majority of target genes were assigned to the same cluster in every sample within the CD19-28z/HA-28z CAR T cell subsets analyzed by EnrichTF/PECA2.

Dataset S3. (separate file)

TF module assignment for clustered TF/target gene interactions called by EnrichTF/PECA2. Each TF identified in the interaction matrix fell into one of two TF/TG modules by hierarchical clustering. The vast majority of TFs were assigned to the same cluster in every sample within the CD19-28z/HA-28z CAR T cell subsets analyzed by EnrichTF/PECA2.

Dataset S4. (separate file)

Significantly differential HiChIP loops between Day 10 CD19-28z and HA-28z CAR T cells. For each differential loop, genomic coordinates for both anchor point windows, the raw contact count within each anchor window, fold-change between CD19-28z and HA-28z loops corresponding to those coordinates, the p-value, and the false-discovery rate are provided.
